# Antinuclear antibodies and mortality in the National Health and Nutrition Examination Survey (1999-2004)

**DOI:** 10.1371/journal.pone.0185977

**Published:** 2017-10-09

**Authors:** Gregg E. Dinse, Christine G. Parks, Clarice R. Weinberg, Helen C. S. Meier, Caroll A. Co, Edward K. L. Chan, Frederick W. Miller

**Affiliations:** 1 Social & Scientific Systems, Durham, North Carolina, United States of America; 2 Epidemiology Branch, National Institute of Environmental Health Sciences, National Institutes of Health, Research Triangle Park, North Carolina, United States of America; 3 Biostatistics and Computational Biology Branch, National Institute of Environmental Health Sciences, National Institutes of Health, Research Triangle Park, North Carolina, United States of America; 4 Joseph J. Zilber School of Public Health, University of Wisconsin-Milwaukee, Milwaukee, Wisconsin, United States of America; 5 University of Florida Health Science Center, Gainesville, Florida, United States of America; 6 Clinical Research Branch, National Institute of Environmental Health Sciences, National Institutes of Health, Bethesda, Maryland, United States of America; University of Alabama at Birmingham, UNITED STATES

## Abstract

**Objective:**

Recent studies suggest antinuclear antibodies (ANA) may be related to mortality risk, but evidence is sparse and inconclusive. Thus, we investigated ANA associations with all-cause and cause-specific mortality in U.S. adults.

**Methods:**

Our sample included 3357 adults (ages ≥20 years) from the 1999–2004 National Health and Nutrition Examination Survey with ANA measurements (1:80 dilution) and mortality data through 2011 (median follow-up: 9.4 years). We estimated hazard ratios (HRs) and 95% confidence intervals (CIs) via weighted Cox regression to assess ANA associations with mortality from all causes, cardiovascular disease (CVD), and cancer. Models adjusted for age, sex, race/ethnicity, education, and obesity. Analyses examined mortality in the full sample and in subgroups based on self-reported histories of CVD and cancer, both overall and stratified by sex and age at enrollment.

**Results:**

Overall, ANA were not strongly associated with death from all causes (HR: 1.13; CI: 0.79, 1.60), from CVD (HR: 1.60; CI: 0.80, 3.20), or from cancer (HR: 1.58; CI: 0.75, 3.33), though all three HR estimates exceeded 1. In the subgroup with a history of cancer, ANA were associated with elevated all-cause mortality in men (HR: 2.28; CI: 1.01, 5.14) and in participants who enrolled at age ≥75 years (HR: 1.99; CI: 1.04, 3.80).

**Conclusion:**

These findings suggest that ANA are not strongly associated with mortality in the general population. Longitudinal studies with repeated assessments are needed to understand the temporal relationship between ANA, aging-associated diseases, and mortality.

## Introduction

Antinuclear antibodies (ANA) are a marker of self-reactivity seen across multiple autoimmune diseases [1–2]. Together with specific autoantibodies, ANA may precede the development of autoimmune disease by years [2–4]. However, ANA are also found in about 12–16% of the general U.S. population and their prevalence increases with age in both men and women [5]. In the 1999–2004 population-representative National Health and Nutrition Examination Survey (NHANES), the prevalence of ANA at ages ≥70 years was nearly double that at ages 12–19 years [5]. Higher ANA prevalence in older adults was also reported in other studies [6–8], though not all studies have shown this pattern [9–11].

The reasons for elevated ANA in healthy and aging individuals are not known, but may include genetic or environmental factors influencing immune regulation and activity [12–14]. Community-based studies have provided limited evidence of ANA associations with non-autoimmune, chronic diseases [6,9,15], though occasionally ANA have been reported to be associated with heart disease, cancer, infections, and certain medications [16–19], often in studies of select clinical samples. Inflammation-related exposures, such as obesity and smoking, have not been associated with higher ANA prevalence in the U.S. population [5].

Despite the increased prevalence of ANA in those individuals who later develop clinical autoimmune diseases, such as systemic lupus erythematosus [3], the consequences of ANA in the general population are not known. Evidence of an association between ANA and mortality is mixed. Two studies reported that ANA were associated with increased all-cause mortality [20–21], but this association was not confirmed in other studies [2,22]. The literature on ANA and cause-specific mortality is sparse and inconclusive. One study reported that ANA were associated with an increased risk of death from cardiovascular disease (CVD) [21]; another study found no ANA association with cancer prevalence but did not report on cancer mortality [2].

In a representative sample of the general U.S. population, we sought to examine associations of ANA with all-cause mortality, as well as mortality specifically from CVD and cancer, the two most common primary (not contributing) causes of death in adult NHANES participants. We examined mortality in all participants and in subgroups with and without a self-reported history of CVD or cancer. Our mortality analyses adjusted for age, sex, race/ethnicity, education, and obesity.

## Participants and methods

### Study participants

We analyzed a subsample of NHANES data collected from 1999 to 2004 (available at http://www.cdc.gov/nchs/nhanes/nhanes_questionnaires.htm). Briefly, the NHANES used a multistage strategy to select a nationally representative sample; a representative subsample of 7106 participants at least 12 years old was then selected for a substudy assessing serum levels of organochlorines, and of these, 4754 participants gave permission for sera storage and had samples available for ANA analysis. Sampling weights were appropriately revised to account for participation in the serum collection substudy. For details on the weights, see the NHANES web site (https://www.cdc.gov/nchs/tutorials/nhanes/SurveyDesign/Weighting/intro.htm). The NHANES collected extensive self-reported information on sociodemographic and health-related factors. Constructed variables such as body mass index (BMI, weight/(height squared)) were also included [23]. The NHANES protocol was approved by the human subjects Institutional Review Board of the U.S. Centers for Disease Control and Prevention and written informed consent was obtained from all participants.

The NHANES data were linked to the National Death Index, which for this analysis provided follow-up information on mortality through December 31, 2011 for participants ≥18 years old (http://www.cdc.gov/nchs/data-linkage/mortality.htm). Data on most health conditions were only available for participants ≥20 years old. Therefore, we excluded the 1190 participants under age 20 years and another 5 participants with missing mortality data. We also excluded 202 women who reported being pregnant, as their physiology and later outcomes could be different [24–25], which left a subsample of 3357 participants for our primary analysis. Except for age at enrollment and pregnancy status, we found no differences in demographics compared with the initial sample after making these exclusions (not shown). All 3357 remaining participants had complete data on enrollment age, sex, race/ethnicity, ANA, and mortality. Adjusted analyses additionally excluded 6 participants missing data on education and 9 participants missing data on BMI. Twenty-one participants missing data on CVD history and 5 participants missing data on cancer history were excluded in the corresponding disease-subgroup analyses.

### Determination of ANA status

Standard indirect immunofluorescence was used to measure ANA in serum specimens, based on commercial HEp-2 ANA slides (Inova Diagnostics) with 1:80 dilutions of sera and staining with DyLight 488-conjugated donkey anti-human IgG antibodies (Jackson ImmunoResearch) as previously reported [5]. Staining intensities were graded from 0 to 4 relative to a standard reference gallery, with values of 3 and 4 considered indicative of ANA positivity [5]. Two independent raters agreed on >95% of the readings for overall intensity ratings, and differences were resolved by consensus or adjudicated by a third rater. Specific autoantibodies were identified in ANA-positive participants, using immuno-precipitation of ^35^S-methionine labeled K562 cell extracts as previously described [5]. These specific autoantibodies were classified as extractable nuclear antigens (ENA) and included: Sjögren's syndrome antigen A (Ro), Sjögren's syndrome antigen B (La), Argonaute 2 (Su), U1 ribonucleoprotein (U1RNP), Smith antigen (Sm), Topoisomerase I, Ribosomal proteins or RNA, Replication Protein A (RPA), isoleucyl-transfer RNA synthetase (OJ), Nucleolar organizer region 90kDa antigen or Upstream binding protein (NOR90), histidyl-transfer RNA synthetase (Jo), threonyl-transfer RNA synthetase (PL-7), alanyl-transfer RNA synthetase (PL-12), glycyl-transfer RNA synthetase (EJ), signal recognition particle (SRP), p70/p80 antigen that is a DNA-binding protein (Ku), Polymyositis-scleroderma antigen (PM-Scl), chromodomain helicase DNA binding protein 4 (Mi-2), RNA polymerase, and U3 ribonucleoprotein (U3RNP). Repeat testing of a random sample showed >98% concordance in intensity rating and antibody specificities.

### Covariates

Previous studies showed greater ANA prevalence in older adults, women, non-Hispanic blacks, and participants neither overweight nor obese [5,26]. In our study sample, these factors were also predictors of mortality, and thus our analyses adjusted for them. Age was treated as a continuous variable; race/ethnicity was categorized as non-Hispanic white, non-Hispanic black, or other; and BMI was categorized as obese (≥30 kg/m^2^), overweight (25 to <30 kg/m^2^), or neither (<25 kg/m^2^). The underweight category of BMI (<18.5 kg/m^2^) was uncommon (N = 59) and did not differ significantly from the healthy category (18.5 to <25 kg/m^2^) with respect to ANA prevalence, so we combined them to form the “neither overweight nor obese” category. As a proxy for socioeconomic status, which was an NHANES sampling-design factor, we included a covariate for education, categorized as less than a high school diploma, a high school diploma, or more than a high school diploma.

### Health conditions

We assessed data on five major health conditions for which self-reported information was available: CVD, cancer, thyroid problems, rheumatoid arthritis, and diabetes. Thyroid problems included goiter and thyroid disease; cancer excluded non-melanoma skin cancer; and CVD included coronary heart disease (CHD), congestive heart failure, and stroke. The CHD subcategory of CVD included anyone diagnosed with CHD, a heart attack, angina, or taking anti-angina medication. Our analysis focused on CVD and cancer because they were the most common causes of death listed. Some instances of thyroid problems, rheumatoid arthritis, and Type 1 diabetes may have an autoimmune etiology, so participants who reported a history of any of these conditions were excluded in a sensitivity analysis. Questions about other specific autoimmune diseases were not included in these cycles of the NHANES. Participants diagnosed with diabetes before age 30 years and taking insulin were classified as having Type 1 diabetes; other diabetics were classified as having Type 2 diabetes [27–29].

### Mortality

We investigated both all-cause mortality and cause-specific mortality. Our analysis of all-cause mortality focused on death due to natural (i.e., non-accidental) causes, as we assumed there was no biologic relationship between ANA and accidental death. Our analysis of cause-specific mortality focused on death due to CVD or cancer, and only if that disease was listed as a primary cause on the death certificate.

### Statistical analyses

In cross-sectional analyses, we applied logistic regression [30] to assess ANA associations with baseline sample characteristics, adjusted only for age at enrollment and NHANES sampling weights. For a detailed description of these weights, see the NHANES web site (https://www.cdc.gov/nchs/tutorials/nhanes/SurveyDesign/Weighting/intro.htm). The age-at-enrollment adjustment used a restricted cubic spline [31] with knots at 32, 43, 55 and 69 years, which were the 20^th^, 40^th^, 60^th^ and 80^th^ percentiles of the age-at-enrollment distribution in the full sample. The ANA association with each characteristic was summarized with an odds ratio (OR) and a 95% confidence interval (CI).

In our mortality analyses, we applied Cox proportional-hazards regression [32] to assess associations between baseline ANA and subsequent death, with age as the time scale and with adjustments for the NHANES sampling weights. Each association between ANA and mortality was summarized with a hazard ratio (HR) and a 95% CI estimated under a covariate-adjusted Cox model for age at death, with baseline ANA status as the predictor of interest. First, we examined associations between all-cause mortality and each individual covariate, with no adjustments for the other covariates. Next, we performed covariate-adjusted evaluations of ANA associations with all-cause mortality in all participants and in subgroups who reported a history of CVD, cancer, or neither disease at baseline. We also investigated ANA associations with cause-specific mortality from CVD or cancer in all participants and in subgroups with a history (or no history) of the target disease at baseline.

Age at death for a participant who died from any non-accidental cause was treated as an uncensored event time in the Cox analysis of all-cause mortality. Similarly, age at death for a participant who died from CVD or cancer was treated as an uncensored event time in the cause-specific analysis of CVD or cancer mortality, respectively. In all mortality analyses, the last known age of a person who did not die from natural (i.e., non-accidental) causes by the end of follow-up was treated as a non-informatively right-censored event time, as was the age at death from any natural cause other than the disease of interest in the cause-specific mortality analyses.

Our mortality analyses accounted for age in several ways: age at death was the outcome of interest, age at risk was a predictor entering the model through the underlying hazard function, and age at enrollment was the left-truncation point signaling when a person became “at risk” of death. We also performed secondary analyses stratified by sex or enrollment age (<75 versus ≥75 years); we selected the age cutoff so that half of the deaths occurred in each stratum. Our covariate-adjusted Cox analyses included race/ethnicity, education, and BMI as covariates; sex was included as a covariate in analyses that did not stratify by sex; and self-reported history of the target disease (CVD or cancer) was included as a covariate in the cause-specific analysis of all participants with respect to death from the target disease.

Several sensitivity analyses were conducted. One examined the impact of excluding participants with ENA or a possible autoimmune disease (i.e., thyroid problems, rheumatoid arthritis or Type 1 diabetes), as autoimmune disease may have influenced mortality differently, via medication use or by other means, compared to those with ANA but without autoimmune disease. Another excluded the first two years of follow-up to reduce possible reverse causality from severe illness or other potential causes of imminent death. A third used a cutoff of 65 years rather than 75 years for the age-at-enrollment strata to investigate a dichotomy common in aging research, which allowed a greater focus on premature deaths.

We used the SURVEYLOGISTIC procedure in SAS (version 9.3, SAS Institute) to perform the logistic regression analyses and the SURVIVAL procedure in SUDAAN (version 11.0.1, Research Triangle Institute) to perform the Cox regression analyses. Both procedures adjusted for the weights, clusters, and strata involved in the complex sampling design. We call a result statistically significant if the corresponding 95% CI does not contain 1. Although many analyses were performed, we present results uncorrected for multiple comparisons.

## Results

Of the 3357 NHANES participants in the study sample, 495 (14.8%) were ANA positive at baseline and 512 (15.3%) died from non-accidental causes (Table 1). The median follow-up time was 9.4 years and the total follow-up was 30,563 person-years. Age-adjusted ORs and their 95% CIs suggest that ANA prevalence was higher in women and non-Hispanic blacks, and was lower in participants who were overweight or obese. Unadjusted HRs and their 95% CIs suggest that all-cause mortality was higher in non-Hispanic blacks and was lower in women, participants with greater than a high school education, and participants who were overweight.

**Table 1:** Baseline sample characteristics, their age-adjusted associations with ANA prevalence, and their unadjusted associations with all-cause mortality.

Characteristic / Category	N (%)	ANA Prevalence		All-Cause Mortality	
		N^+^ (%)	OR (95% CI) ^a^	D (%)	HR (95% CI) ^b^
All Participants	3357 (100.0)	495 (14.8)	not applicable	512 (15.3)	not applicable
Age at Enrollment (years)					
20–74	2919 (87.0)	409 (14.0)	1.00 (referent)	257 (8.8)	-----^c^
≥75	438 (13.1)	86 (19.6)	1.49 (0.72, 3.07)	255 (58.2)	-----^c^
Sex					
Men	1668 (49.7)	180 (10.8)	1.00 (referent)	290 (17.4)	1.00 (referent)
Women	1689 (50.3)	315 (18.7)	2.22 (1.69, 2.91)	222 (13.1)	0.59 (0.50, 0.69)
Race/Ethnicity					
Non-Hispanic White	1740 (51.8)	257 (14.8)	1.00 (referent)	314 (18.1)	1.00 (referent)
Non-Hispanic Black	597 (17.8)	101 (16.9)	1.26 (0.94, 1.69)	87 (14.6)	1.46 (1.21, 1.77)
Other	1020 (30.4)	137 (13.4)	0.90 (0.64, 1.27)	111 (10.9)	0.95 (0.80, 1.13)
Education ^d^					
< High School Diploma	1094 (32.7)	167 (15.3)	1.00 (referent)	238 (21.8)	1.00 (referent)
High School Diploma	772 (23.0)	109 (14.1)	1.01 (0.74, 1.38)	112 (14.5)	0.90 (0.72, 1.12)
> High School Diploma	1485 (44.3)	216 (14.6)	0.98 (0.75, 1.29)	159 (10.7)	0.74 (0.62, 0.88)
BMI ^e^					
Not Overweight or Obese	1112 (33.2)	187 (16.8)	1.00 (referent)	186 (16.7)	1.00 (referent)
Overweight	1192 (35.6)	156 (13.1)	0.75 (0.56, 0.98)	181 (15.2)	0.76 (0.61, 0.95)
Obese	1044 (31.2)	150 (14.4)	0.77 (0.60, 1.00)	141 (13.5)	0.94 (0.73, 1.22)

Abbreviations: ANA = antinuclear antibodies; OR = odds ratio; CI = confidence interval; HR = hazard ratio; N (%) = number (percent) of participants in category of interest; N+ (%) = category-specific number (percent) who were ANA positive at baseline; D (%) = category-specific number (percent) who died from non-accidental causes; BMI = body mass index.

^a^ Each OR estimate was derived from a logistic model for ANA prevalence; the only covariates were the characteristic of interest and a restricted cubic spline in continuous age at enrollment. Participants with missing values were excluded.

^b^ Each HR estimate was derived from a Cox model for all-cause mortality. The continuous event time variable for all-cause mortality was age at death from non-accidental causes and the only covariate was the characteristic of interest. Participants with missing values were excluded.

^c^ The time scale for the Cox analysis was age, which already finely adjusted for age at risk via the baseline hazard function; thus, we did not fit an all-cause mortality model with age at enrollment as a covariate.

^d^ There were 6 participants excluded due to missing information on education.

^e^ There were 9 participants excluded due to missing information on BMI. Categories for “Underweight” (with 59 participants, or 2%) and “Healthy” (with 1053 participants, or 31%) were combined to form the “Not Overweight or Obese” category. The three categories are <25 kg/m^2^ (Not Overweight or Obese), 25 to <30 kg/m^2^ (Overweight), and ≥30 kg/m^2^ (Obese)

In covariate-adjusted analyses, we first investigated associations between baseline ANA and all-cause mortality, in all participants and in subgroups with and without a history of CVD or cancer. We did not observe a statistically significant ANA association with all-cause mortality, either overall (HR: 1.13; CI: 0.79, 1.60) or in sex or enrollment-age strata (Table 2); participants who enrolled at age ≥75 years had the largest estimate (HR: 1.27; CI: 0.89, 1.82). In participants with a history of cancer, all-cause mortality was modestly but not statistically significantly associated with ANA (HR: 1.38; CI: 0.67, 2.82). This association was stronger (and statistically significant) in men with a history of cancer (HR: 2.28; CI: 1.01, 5.14) and in participants who enrolled at age ≥75 years with a history of cancer (HR: 1.99; CI: 1.04, 3.80). However, these estimates are based on relatively few relevant events (of the 29 ANA-positive participants with a history of cancer, only 5 deaths occurred in men and 8 in participants who enrolled at age ≥75 years). In participants with a history of CVD, and in those with no history of CVD or cancer, ANA status was not significantly associated with all-cause mortality.

**Table 2:** Covariate-adjusted associations between baseline ANA status and all-cause mortality within selected groups.

Group Analyzed	Summary	All Participants	Sex		Age at Enrollment (years)	
			Men	Women	20–74	≥75
All Participants	HR^a^	1.13	1.12	1.11	1.01	1.27
	95% CI	(0.79, 1.60)	(0.66, 1.93)	(0.74, 1.67)	(0.60, 1.71)	(0.89, 1.82)
	N/D/D^+^	3342/505/91	1661/287/37	1681/218/54	2911/256/38	431/249/53
History of CVD	HR^a^	0.93	0.82	1.06	0.59 ^b^	1.29
	95% CI	(0.59, 1.49)	(0.37, 1.80)	(0.62, 1.84)	(0.24, 1.48) ^b^	(0.76, 2.18)
	N/D/D^+^	369/156/32	206/93/12	163/63/20	237/61/9	132/95/23
History of Cancer	HR^a^	1.38	2.28 ^b^	1.44 ^b^	-----^c^	1.99 ^b^
	95% CI	(0.67, 2.82)	(1.01, 5.14) ^b^	(0.39, 5.32) ^b^	-----^c^	(1.04, 3.80) ^b^
	N/D/D^+^	226/88/11	103/55/5	123/33/6	149/34/3	77/54/8
No history of CVD or cancer	HR^a^	1.11	1.23	0.96	1.10	1.16
	95% CI	(0.75, 1.63)	(0.65, 2.35)	(0.55, 1.67)	(0.62, 1.96)	(0.78, 1.74)
	N/D/D^+^	2773/278/50	1367/150/20	1406/128/30	2536/163/25	237/115/25

Abbreviations: ANA = antinuclear antibodies; HR = hazard ratio; CI = confidence interval; N = number of participants analyzed; D = number of participants who died; D+ = number of ANA-positive participants who died; CVD = cardiovascular disease; BMI = body mass index.

^a^ Each HR estimate was derived from a Cox model for all-cause mortality, fitted within a given group of participants and focused on the effect of baseline ANA status on death from non-accidental causes. The continuous event time variable for all-cause mortality was age at death from non-accidental causes and baseline ANA status was represented by a dichotomous indicator variable. All analyses adjusted for sex (except the separate analyses of men and women), race/ethnicity, education, and BMI. Participants with missing values were excluded.

^b^ Interpret cautiously, as these results are based on fewer than 10 events of interest.

^c^ Results are not shown because they are based on fewer than 5 events of interest.

We also investigated ANA associations with CVD mortality and cancer mortality. Covariate-adjusted HRs and their 95% CIs did not show statistically significant ANA associations with death due to CVD or cancer, either among all participants or in subgroups with or without a baseline history of the disease of interest (Table 3). Although the overall association between ANA and CVD mortality (HR: 1.60; CI: 0.80, 3.20) was not statistically significant, the HR estimates were consistently elevated across both sexes and both age-at-enrollment strata, with the largest effect estimates in men (HR: 1.70; CI: 0.57, 5.07) and participants who enrolled at age ≥75 years (HR: 1.69; CI: 0.93, 3.07). Similarly, although the overall ANA association with cancer mortality (HR: 1.58; CI: 0.75, 3.33) was not statistically significant, the estimated HRs were approximately the same magnitude across all sex and age-at-enrollment strata.

**Table 3:** Covariate-adjusted associations between baseline ANA status and cause-specific mortality within selected groups.

Cause of Death / Group Analyzed	Summary	All Participants	Sex		Age at Enrollment (years)	
			Men	Women	20–74	≥75
CVD						
All participants	HR ^a^	1.60	1.70	1.37	1.44^b^	1.69
	95% CI	(0.80, 3.20)	(0.57, 5.07)	(0.66, 2.84)	(0.38, 5.48)^b^	(0.93, 3.07)
	N/C/C^+^	3315/94/20	1653/54/10	1662/40/10	2891/42/7	424/52/13
History of CVD	HR ^a^	1.74^b^	1.11^b^	-----^c^	-----^c^	2.32^b^
	95% CI	(0.68, 4.45)^b^	(0.32, 3.82)^b^	-----^c^	-----^c^	(0.79, 6.76)^b^
	N/C/C^+^	366/39/9	205/26/5	161/13/4	235/20/3	131/19/6
No history of CVD	HR ^a^	1.55	2.92^b^	0.95^b^	-----^c^	1.05^b^
	95% CI	(0.61, 3.96)	(0.73, 11.63)^b^	(0.40, 2.29)^b^	-----^c^	(0.55, 1.99)^b^
	N/C/C^+^	2949/55/11	1448/28/5	1501/27/6	2656/22/4	293/33/7
Cancer						
All participants	HR ^a^	1.58	1.62	1.64	1.62	1.58^b^
	95% CI	(0.75, 3.33)	(0.54, 4.80)	(0.70, 3.84)	(0.71, 3.69)	(0.66, 3.79)^b^
	N/C/C^+^	3331/119/27	1656/70/12	1675/49/15	2903/75/18	428/44/9
History of cancer	HR ^a^	2.11^b^	-----^c^	-----^c^	-----^c^	-----^c^
	95% CI	(0.50, 8.96)^b^	-----^c^	-----^c^	-----^c^	-----^c^
	N/C/C^+^	226/41/6	103/28/2	123/13/4	149/20/3	77/21/3
No history of cancer	HR ^a^	1.46	1.64	1.34	1.55	1.31^b^
	95% CI	(0.74, 2.87)	(0.45, 6.02)	(0.72, 2.52)	(0.75, 3.19)	(0.50, 3.47)^b^
	N/C/C^+^	3105/78/21	1553/42/10	1552/36/11	2754/55/15	351/23/6

Abbreviations: ANA = antinuclear antibodies; HR = hazard ratio; CI = confidence interval; N = number of participants analyzed; C = number of participants who died from the cause of interest; C+ = number of ANA-positive participants who died from the cause of interest; CVD = cardiovascular disease; BMI = body mass index.

^a^ Each HR estimate was derived from a Cox model for cause-specific mortality, fitted within a given group of participants and focused on the effect of baseline ANA status on death from the cause of interest. The continuous event time variable for cause-specific mortality was age at death from the cause of interest (either CVD or cancer) and baseline ANA status was represented by a dichotomous indicator variable. All analyses adjusted for sex (except the separate analyses of men and women), race/ethnicity, education, and BMI. Analyses that did not stratify on disease history also adjusted for self-reported history of the disease of interest at baseline. Participants with missing values were excluded.

^b^ Interpret cautiously, as these results are based on fewer than 10 events of interest.

^c^ Results are not shown because they are based on fewer than 5 events of interest.

Results of several sensitivity analyses are shown in Table 4. In a sensitivity analysis that excluded the 496 participants with ENA or a potential autoimmune disease, the positive ANA association with all-cause mortality was statistically significant in those who enrolled at age ≥75 years (HR: 1.51; CI: 1.03, 2.21), in the subgroup of older enrollees with a history of cancer (HR: 2.14; CI: 1.07, 4.28), and in the subgroup of older enrollees with no history of CVD or cancer (HR: 1.54; CI: 1.01, 2.36). In a second sensitivity analysis that excluded the initial two years of follow-up, the positive ANA association with all-cause mortality was statistically significant in participants who enrolled at age ≥75 years with a history of cancer (HR: 2.64; CI: 1.09, 6.38) and the positive ANA association with CVD mortality was statistically significant in participants who enrolled at age ≥75 years (HR: 1.99; CI: 1.04, 3.78). In a third sensitivity analysis that lowered the cut point for the age-at-enrollment strata by 10 years, the positive ANA association with CVD mortality was statistically significant for participants who enrolled at age ≥65 years (HR: 1.90; CI: 1.05, 3.43) and the positive ANA association with cancer mortality was statistically significant in participants who enrolled at age <65 years (HR: 2.70; CI: 1.02, 7.14).

**Table 4:** Covariate-adjusted ANA associations with mortality by cause of death, group analyzed, and analysis performed.

Cause of Death	Group Analyzed	Analysis Performed	HR (95% CI) ^a^	N/C/C^+^
All Causes	Enrolled at age ≥75 years	Primary Analysis	1.27 (0.89, 1.82)	431/249/53
		Exclude participants with ENA or a possible autoimmune disease	1.51 (1.03, 2.21)	329/185/38
	Enrolled at age ≥75 years,	Primary Analysis	1.99 (1.04, 3.80)^b^	77/54/8
	history of cancer	Exclude first 2 years of follow-up	2.64 (1.09, 6.38)^b^	65/43/7
		Exclude participants with ENA or a possible autoimmune disease	2.14 (1.07, 4.28)^b^	60/42/6
	Enrolled at age ≥75 years,	Primary Analysis	1.16 (0.78, 1.74)	237/115/25
	no history of CVD or cancer	Exclude participants with ENA or a possible autoimmune disease	1.54 (1.01, 2.36)	189/90/19
CVD	Enrolled at age ≥75 years	Primary Analysis	1.69 (0.93, 3.07)	424/52/13
		Exclude first 2 years of follow-up	1.99 (1.04, 3.78)^b^	379/34/9
	Enrolled at age ≥65 years	Redefine age-at-enrollment strata	1.90 (1.05, 3.43)	867/74/18
Cancer	Enrolled at age <75 years	Primary Analysis	1.62 (0.71, 3.69)	2903/75/18
	Enrolled at age <65 years	Redefine age-at-enrollment strata	2.70 (1.02, 7.14)	2454/39/11

Abbreviations: ANA = antinuclear antibodies; HR = hazard ratio; CI = confidence interval; N = number of participants analyzed; C = number of participants who died from the cause of interest; C+ = number of ANA-positive participants who died from the cause of interest; CVD = cardiovascular disease; BMI = body mass index.

^a^ Each HR estimate was derived from a Cox model for cause-specific mortality, fitted within a given group of participants and focused on the effect of baseline ANA status on death from the cause of interest. The continuous event time variable for all-cause mortality was age at death from non-accidental causes, the continuous event time variable for cause-specific mortality was age at death from the cause of interest (either CVD or cancer), and baseline ANA status was represented by a dichotomous indicator variable. All analyses adjusted for age at risk (via the baseline hazard function) and included covariates for sex, race/ethnicity, education, and BMI. Analyses that did not stratify on disease history also adjusted for self-reported history of the disease of interest at baseline. Participants with missing values were excluded.

^b^ Interpret cautiously, as these results are based on fewer than 10 events of interest.

## Discussion

In this general population sample, our primary aim was to assess ANA associations with all-cause mortality and mortality from two major causes of death, CVD and cancer. We did not find a strong association of ANA with all-cause mortality, but possible associations were noted between ANA and increased mortality from CVD and cancer, both overall and across sex and age-at-enrollment strata. Associations between ANA and all-cause mortality were statistically significant in certain subgroups with a history of cancer (i.e., men and participants who enrolled at age ≥75 years). Though suggestive, these results warrant cautious interpretation due to the exploratory nature of subgroup analyses (which were not adjusted for multiple comparisons) and the small number of deaths among ANA-positive participants in those subgroups.

Previous studies investigated associations between ANA and all-cause mortality, with mixed results (Table 5). Like ours, two studies did not find a statistically significant ANA association with all-cause mortality. One was a small study of the elderly [22]; all-cause mortality rates did not significantly differ by ANA status (p = 0.718), though no HR was reported. The other study was closer to ours in size and age range [2]; the authors reported a non-significant ANA association with all-cause mortality (HR: 1.40; CI: 0.94, 2.09). A larger study [20] reported a statistically significant ANA association with all-cause mortality (HR: 1.19, CI: 1.04, 1.35); their estimated effect size was similar to ours (HR: 1.13). Another study, which excluded persons with CVD or autoimmune disease with immunosuppressant use [21], reported statistically significant ANA associations with increased all-cause and CVD mortality, but their results are not directly comparable with ours due to differences in the study samples, the assay used (enzyme-linked immunosorbent assay versus immunofluorescence), and the type of ANA predictor used (continuous log ANA concentration versus dichotomous ANA status).

**Table 5:** Summary of several studies that investigated ANA associations with mortality.

Study	Description	Event Counts	Reported HR (and 95% CI) for ANA
Present Study	Population-based cohort study in US, with follow-up through 2011 (median: 9.4 years).	3357 participants	All-cause
			overall: 1.13 (0.79, 1.60)
	Age: range ≥20 years (73% <65; 87% <75).	512 all-cause deaths	men with cancer: 2.28 (1.01, 5.14)
	Sex: 50% women.		elderly with cancer: 1.99 (1.04, 3.80)
	ANA positive defined as titer ≥80.	94 CVD deaths	
	Cox analysis, adjusted for age, sex, race/ ethnicity, education, and body mass index (cause-specific mortality analyses also adjusted for history of the target disease).		CVD
		119 cancer deaths	overall: 1.60 (0.80, 3.20)
			Cancer
			overall: 1.58 (0.75, 3.33)
Hurme et al. [22]	Population-based cohort study in Finland, with follow-up through 2000 (for 4 years).	284 participants	No HR provided, but authors reported there was no association of ANA with all-cause mortality (p = 0.718).
	Age: range 90–99 years (0% <65; 0% <75).	171 all-cause deaths	
	Sex: 76% women.		
	ANA positive defined as titer ≥160 (similar unreported results for titer ≥80).		
	Cox analysis, adjusted for age and sex.		
Selmi et al. [2]	Population-based retrospective study in Italy, with follow-up through 2013 (for 15 years).	2663 participants	All-cause
			overall: 1.40 (0.94, 2.09)
	Age: range 18–75 years (92% <65).	122 all-cause deaths	
	Sex: 50% women.		
	ANA positive defined as any titer.		
	Cox analysis, adjusted for age and sex.		
	Analysis excluded participants with connective tissue diseases.		
Liang et al. [20]	Population-based cohort study in US, with follow-up through 2007 (mean: 9.2 years).	7852 participants	All-cause
			overall: 1.18 (1.04, 1.34)
	Age: mean 47.5 years (no age range reported).	1142 all-cause deaths	men: 1.17 (0.95, 1.44)
	Sex: 69% women.		women: 1.20 (1.02, 1.41)
	ANA positive defined as titer ≥40 (ANA measured for clinical purposes).		
	Cox analysis, adjusted for age, sex, year, rheumatic disease, and several comorbidities.		
	Analysis excluded first 6 months of follow-up and participants with CVD.		
Solow et al. [21]	Population-based cohort study in US, with follow-up through 2010 (median: 9.4 years).	2803 participants	All-cause
			overall: 1.27 (1.10, 1.46)*
	No age or sex information reported.	158 all-cause deaths	
	ANA measured and reported in ELISA units as a continuous variable.*		CVD
		54 CVD deaths	overall: 1.42 (1.13, 1.77)*
	Cox analysis, adjusted for age, sex, and race/ ethnicity.		
	Analysis excluded persons with CVD or auto-immune disease with immunosuppressant use.		

Abbreviations: ANA = antinuclear antibodies; HR = hazard ratio; CI = confidence interval; CVD = cardiovascular disease; elderly = enrolled at age ≥75 years.

* Continuous covariate used for ANA, and HR results reported per 1 standard deviation increase in log[ANA].

While we saw little evidence of ANA associations with all-cause mortality, regardless of CVD history at baseline, we saw similar ANA associations with CVD mortality overall and across sex and age-at-enrollment strata. However, the observed associations were not statistically significant except in older enrollees; that is, participants who enrolled at age ≥65 years and also those who enrolled at age ≥75 years in an analysis that excluded the first two years of follow-up (Table 4). Other studies showed increased CVD mortality in patients with systemic autoimmune diseases, which are often associated with elevations in autoantibodies such as ANA [33–34]. Reasons for this have not been elucidated, but research has often been directed toward determinants of inflammation and medical treatment [35–37].

Our findings suggest that all-cause mortality was higher in certain subgroups of ANA-positive participants with a history of cancer (i.e., men and older enrollees), but sample sizes were too small to assess associations between ANA and cancer mortality in these subgroups. The observation that ANA associations with mortality appear to increase in older age groups may reflect the increasing dysregulation of the immune system with age, which is also known to impact the ability to eliminate neoplasms [38]. For both men and older enrollees, baseline ANA prevalence was lower in those with a history of cancer than in those with no history of cancer (not shown); this suggests the possibility of survival-related length-biased sampling and provides additional evidence of higher mortality for ANA-positive people with cancer in these subgroups. For example, if ANA-positive men die quickly after getting cancer, they will have a reduced probability of being sampled as ANA-positive men with a history of cancer.

The literature on ANA and cancer is sparse; some studies suggest that ANA may be associated with risk of malignant disease and with decreased survival in those with cancer [39–40], while ANA were shown to predict increased survival in melanoma patients treated with interferon [41]. A growing literature highlights the association between cancers and autoimmune diseases [42–43]. While the mechanisms for this association remain unclear, many tumors express antigens that can induce immune responses, ANAs, and other autoantibodies in some patients [44]. Our present findings require cautious interpretation due to the small number of ANA-positive participants who had a history of cancer and died. Also, data on ANA status and self-reported cancer history were only collected at baseline, so our findings of associations between ANA, cancer history, and death do not necessarily imply causality.

Analyses stratified by major health conditions provided evidence that main effects were independent of baseline disease status. We did not consider cross-sectional ANA associations with diseases as a primary study aim, due to the lack of temporal data on ANA relative to the onset of morbidity. Other common health conditions, such as diabetes and obesity, may contribute to the risk of dying from a variety of diseases. Diabetes was inversely associated with ANA at baseline (not shown), as was being overweight or obese (Table 1). Adjustment for BMI did not meaningfully change our results. Further research on the relationship of diabetes, obesity, and autoimmunity may be warranted given their increased prevalence in the general population.

We consider this research hypothesis-generating, in large part due to the limited body of knowledge about the natural history of increasing ANA prevalence associated with aging, the potential mechanism(s) by which ANA might increase mortality, and whether these mechanisms might be expected to differ by age. Some of our analyses suggested an increased susceptibility to ANA-associated mortality in the elderly. We chose a cut point of 75 years when defining our age-at-enrollment strata, partly to achieve comparability with other studies (e.g., [2]) and partly to have similar numbers of deaths in the two strata. However, because some results varied with how the older age-at-enrollment stratum was defined, choosing an appropriate age cut point might deserve further consideration. Causes of death in the population vary by age and also, to some extent, by sex. For example, the percent of deaths from cancer may in general decrease after age 60 [45], but the trajectory varies by sex [46]. Our findings suggest that additional investigation of age and sex differences in ANA associations with mortality are warranted in a larger study sample.

Our primary aim was to elucidate the mortality implications of having ANA in the general U.S. population. In addition to considering subgroups of participants without a history of CVD or cancer at baseline, we conducted a sensitivity analysis that excluded the first two years of follow-up, to avoid the possibility that ANA levels could be impacted by late stage disease and imminent death. Notably, associations of ANA with mortality persisted in certain subgroups of older enrollees with this modification (Table 4). These findings are consistent with the idea that ANA may be a non-specific marker of immune dysfunction and mortality risk in the elderly.

In sum, prior investigations of ANA associations with mortality had one or more of the following limitations: low power due to a relatively small sample size; selection bias linked to geographic or referral patterns; lack of adjustment for multiple confounders; short follow-up; little information on cause of death; and non-traditional ANA measurement. Our study addressed many of these limitations via covariate-adjusted analyses of ANA associations with all-cause and cause-specific mortality in a large representative sample of U.S. adults. While our study has many strengths, a number of limitations remain, including: exclusion of institutionalized persons in the NHANES survey; examination of only one titer to define ANA positivity due to limited statistical power; low power to assess infrequently reported health conditions and uncommon causes of death; incomplete ascertainment of self-reported diseases; inability to confirm diagnoses via medical record review; and inability to account for transitions from ANA-negative to ANA-positive (or vice versa) or from absence to presence of diagnosed disease (or vice versa) during the long follow-up time. We also used a non-standard definition of all-cause mortality by focusing on natural (i.e., non-accidental) deaths; however, only 12 deaths were classified as accidental and none of those participants were ANA positive. Redefining all-cause mortality to include accidental deaths led to nearly identical results (not shown).

Despite limitations, this large study addressed several important knowledge gaps using a representative sample of the adult U.S. population. Many of our findings with respect to ANA associations with all-cause and CVD mortality were consistent with those previously reported in the literature, and we also generated new hypotheses regarding ANA associations with cancer mortality as well as with all-cause mortality among individuals with a history of cancer.
